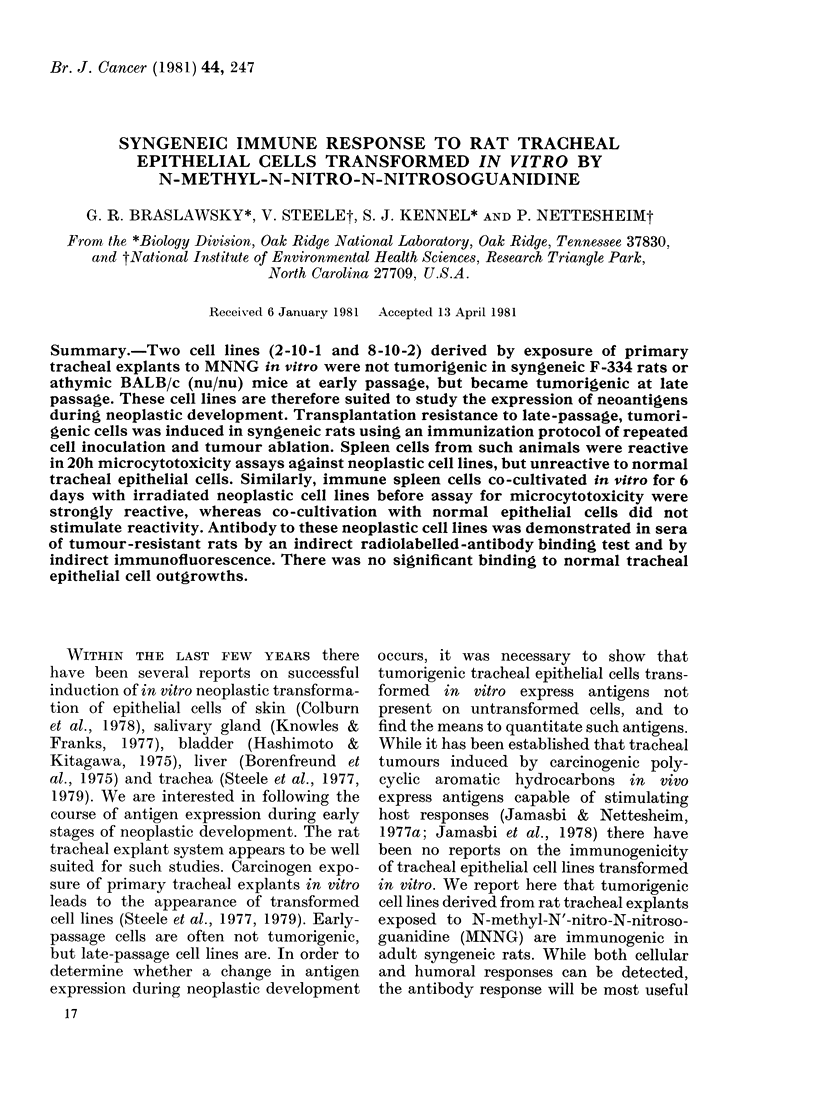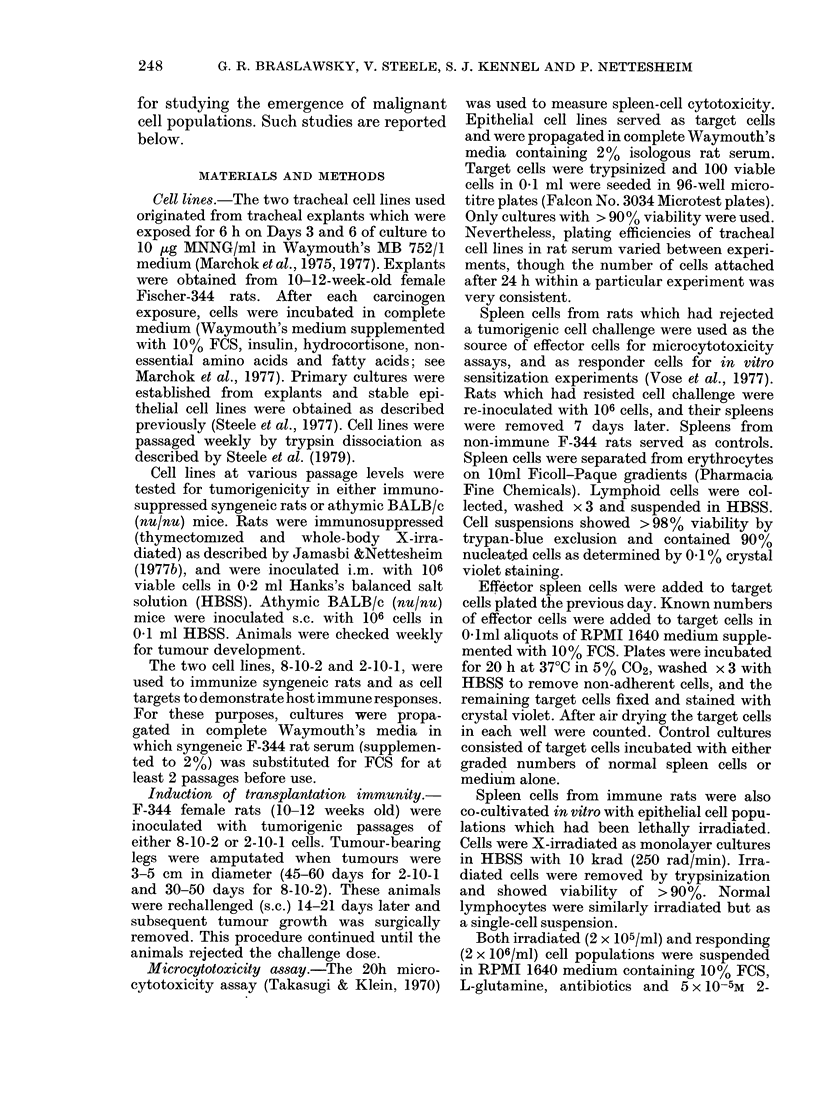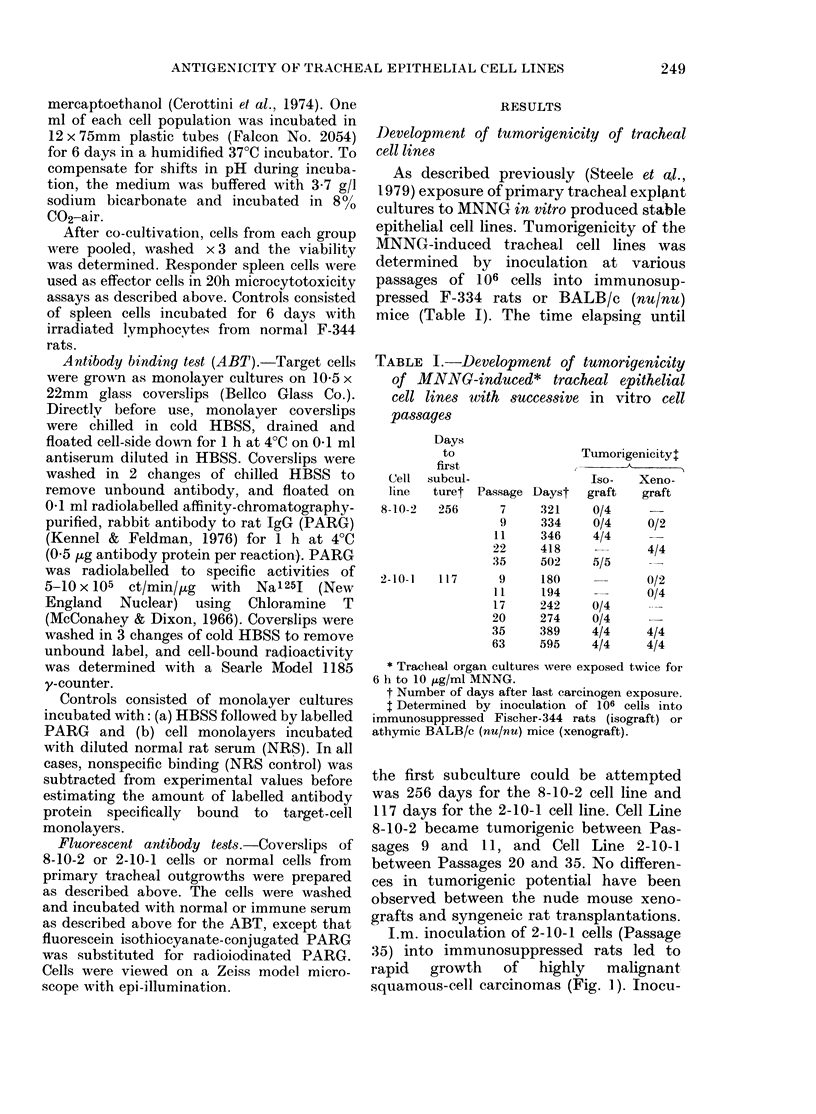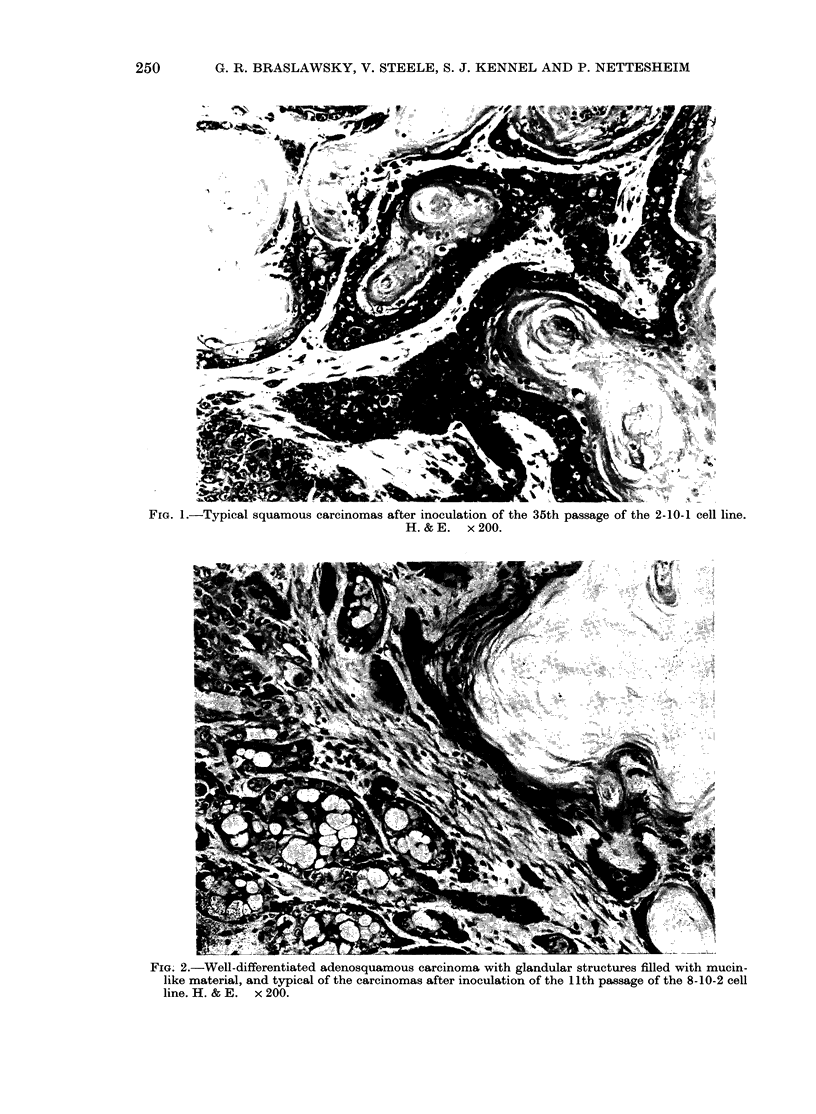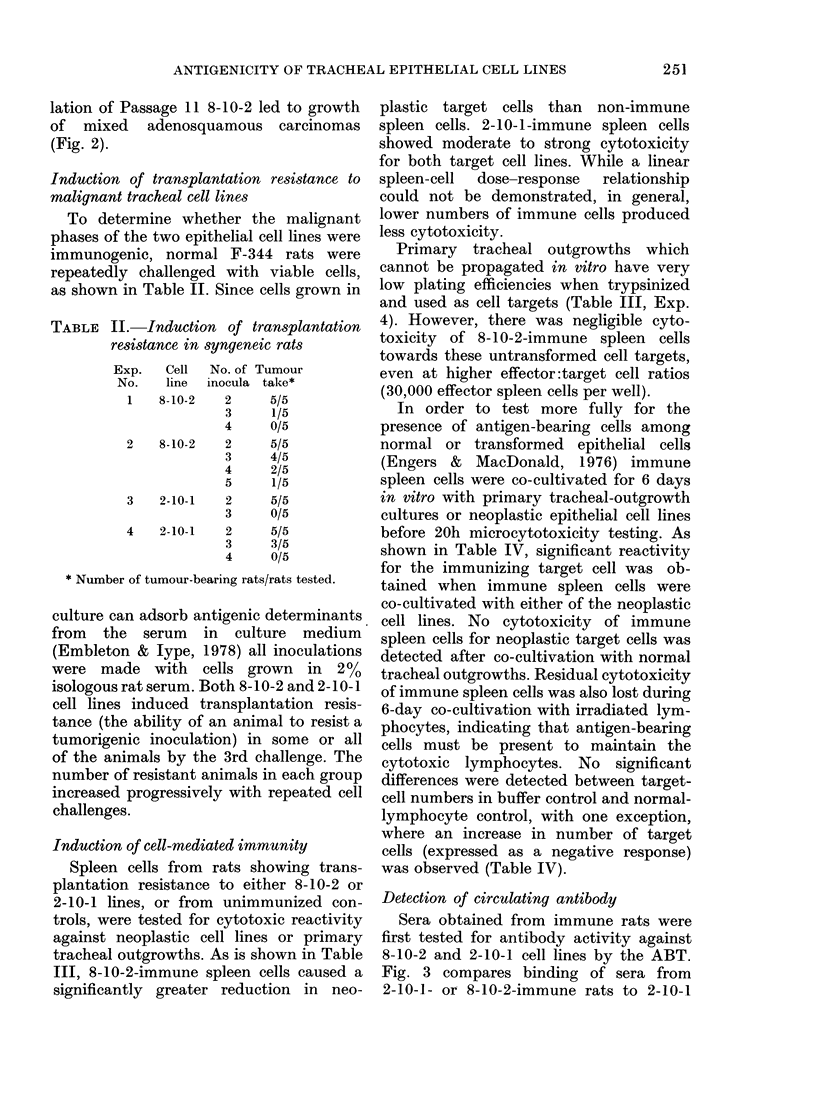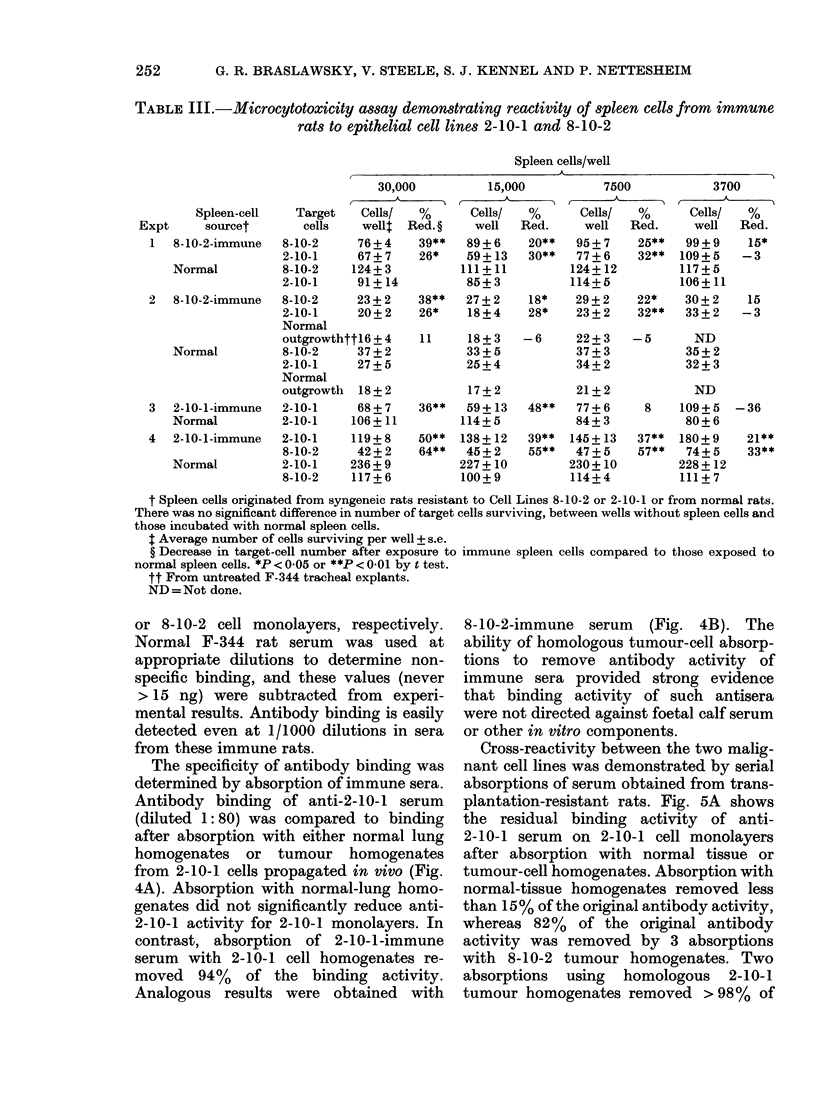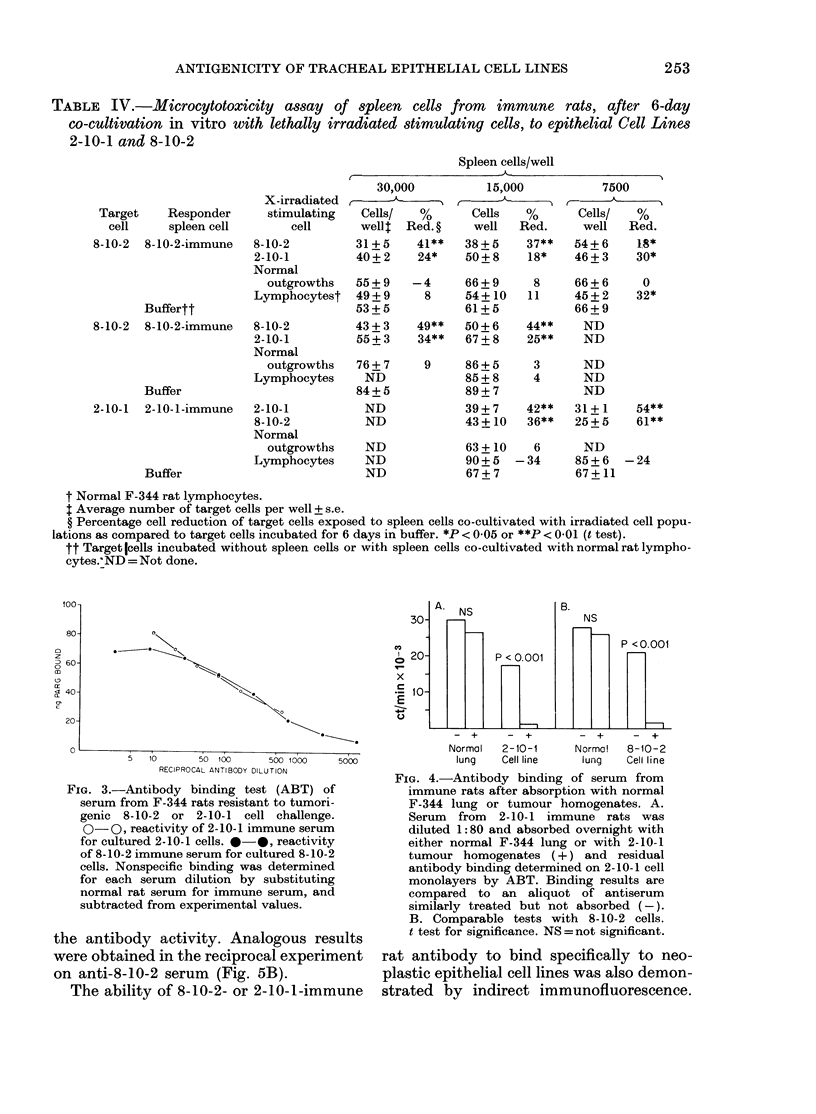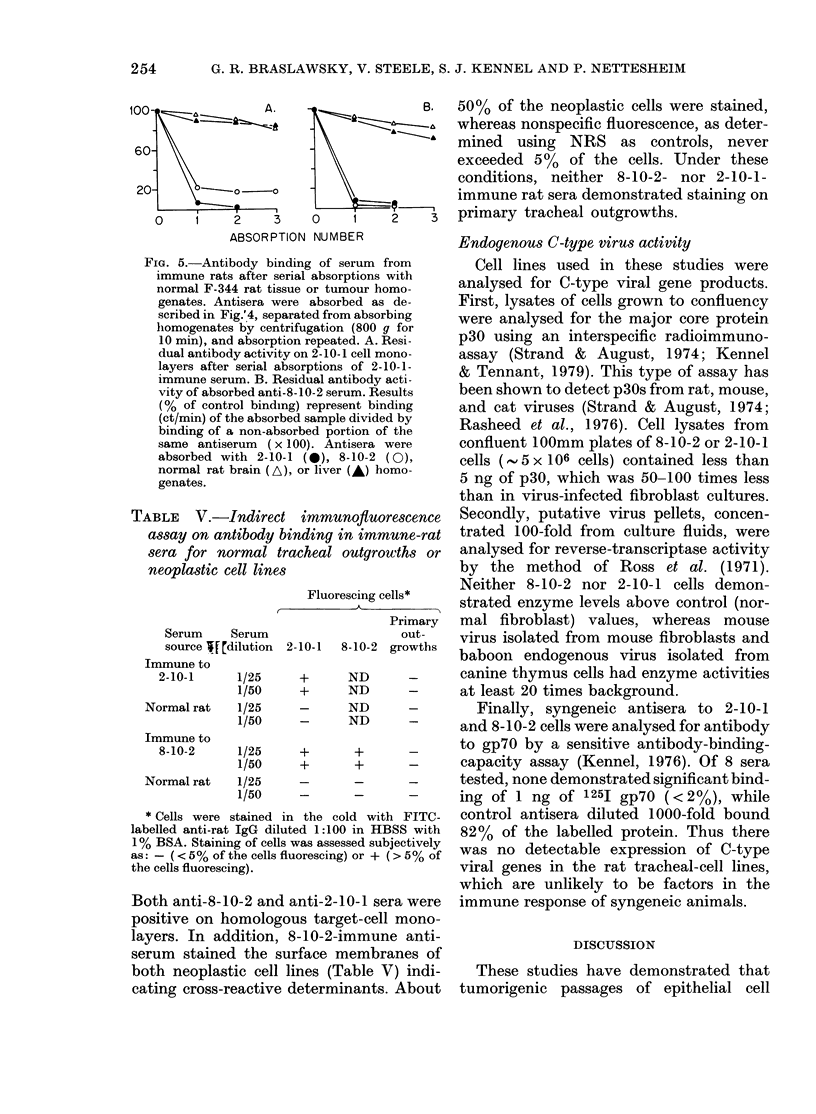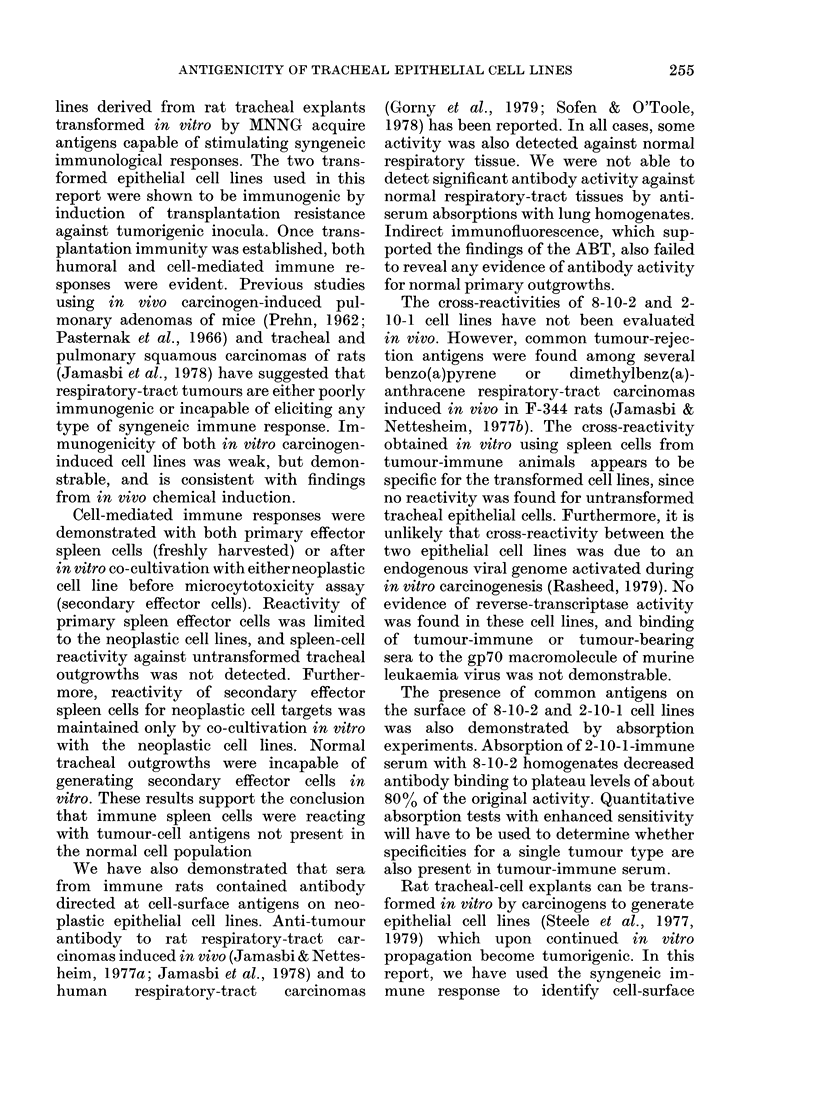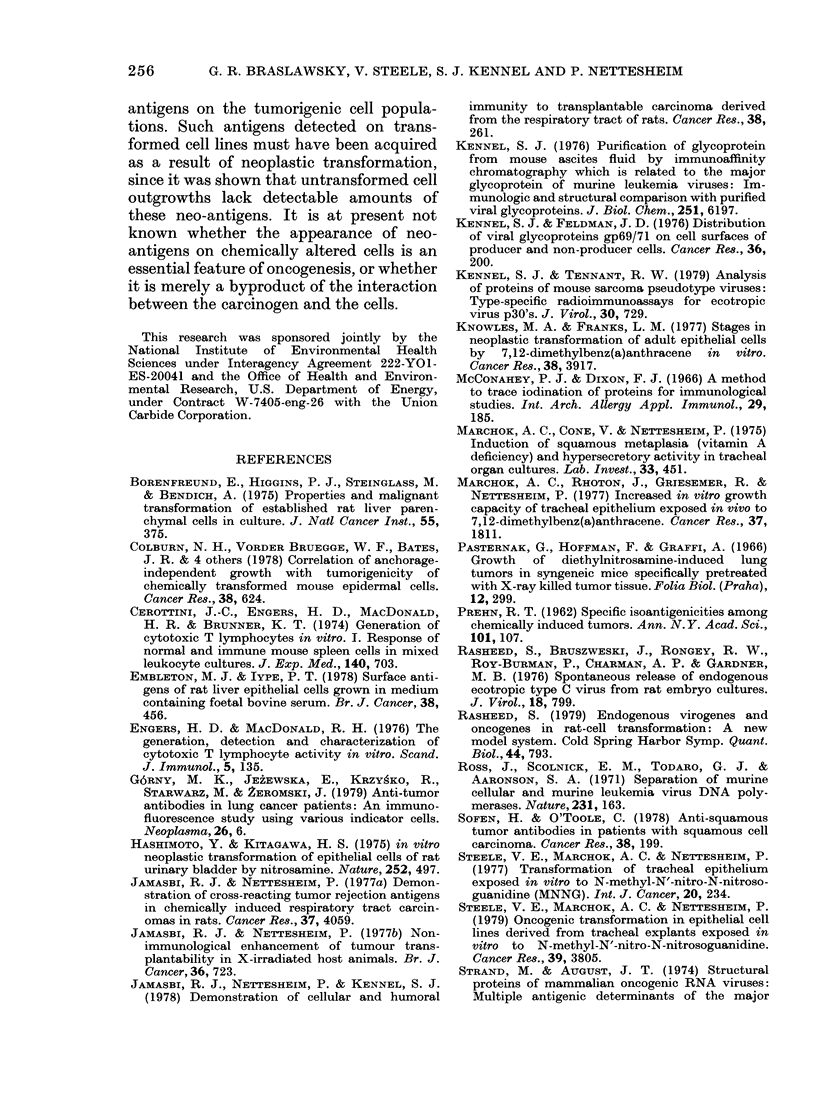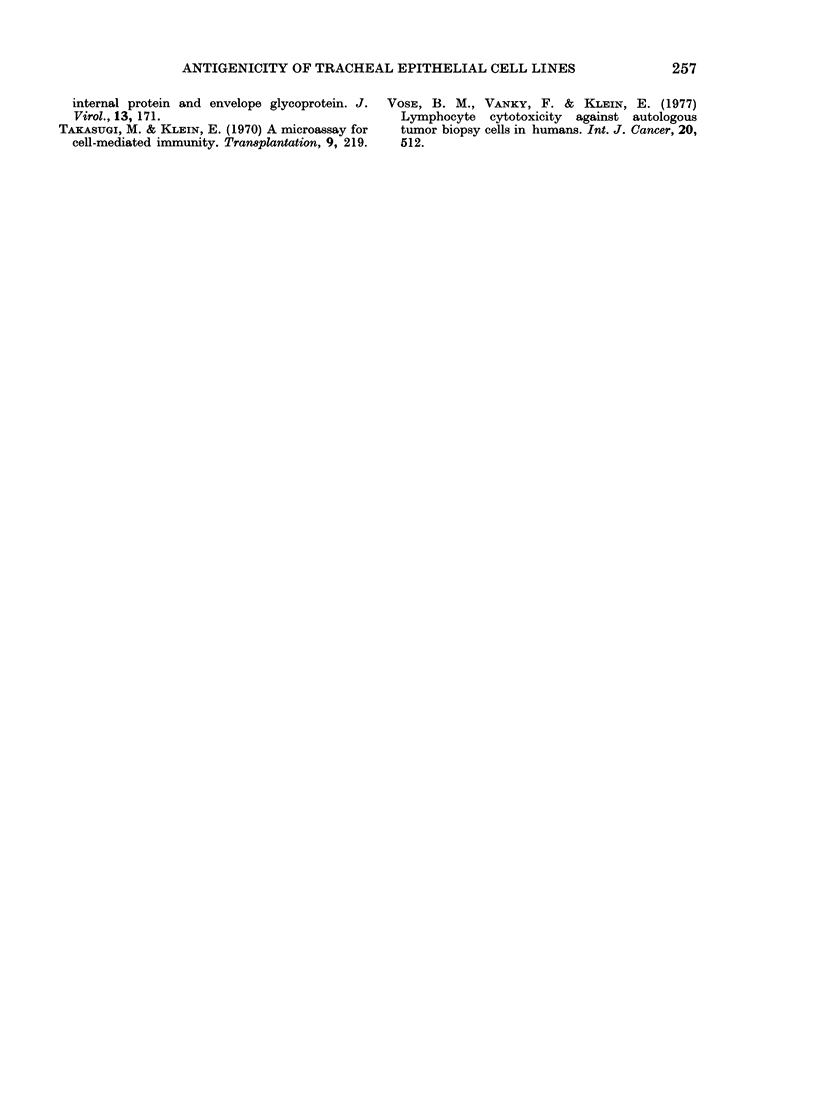# Syngeneic immune response to rat tracheal epithelial cells transformed in vitro by N-methyl-N-nitro-N-nitrosoguanidine.

**DOI:** 10.1038/bjc.1981.176

**Published:** 1981-08

**Authors:** G. R. Braslawsky, V. Steele, S. J. Kennel, P. Nettesheim

## Abstract

**Images:**


*Br. J. Cancer* (1981) **44**, 247

### SYNGENEIC IMMUNE RESPONSE TO RAT TRACHEAL EPITHELIAL CELLS TRANSFORMED *IN VITRO* BY N-METHYL-N-NITRO-N-NITROSOGUANIDINE

G. R. BRASLAWSKY*, V. STEELE†, S. J. KENNEL* AND P. NETTESHEIM†

*From the *Biology Division, Oak Ridge National Laboratory, Oak Ridge, Tennessee* 37830, *and †National Institute of Environmental Health Sciences, Research Triangle Park, North Carolina* 27709, *U.S.A.*

Received 6 January 1981 Accepted 13 April 1981

**Summary.—Two cell lines (2-10-1 and 8-10-2) derived by exposure of primary tracheal explants to MNNG *in vitro* were not tumorigenic in syngeneic F-334 rats or athymic BALB/c (nu/nu) mice at early passage, but became tumorigenic at late passage. These cell lines are therefore suited to study the expression of neoantigens during neoplastic development. Transplantation resistance to late-passage, tumorigenic cells was induced in syngeneic rats using an immunization protocol of repeated cell inoculation and tumour ablation. Spleen cells from such animals were reactive in 20h microcytotoxicity assays against neoplastic cell lines, but unreactive to normal tracheal epithelial cells. Similarly, immune spleen cells co-cultivated *in vitro* for 6 days with irradiated neoplastic cell lines before assay for microcytotoxicity were strongly reactive, whereas co-cultivation with normal epithelial cells did not stimulate reactivity. Antibody to these neoplastic cell lines was demonstrated in sera of tumour-resistant rats by an indirect radiolabelled-antibody binding test and by indirect immunofluorescence. There was no significant binding to normal tracheal epithelial cell outgrowths.**

WITHIN THE LAST FEW YEARS there have been several reports on successful induction of *in vitro* neoplastic transformation of epithelial cells of skin (Colburn *et al.*, 1978), salivary gland (Knowles & Franks, 1977), bladder (Hashimoto & Kitagawa, 1975), liver (Borenfreund *et al.*, 1975) and trachea (Steele *et al.*, 1977, 1979). We are interested in following the course of antigen expression during early stages of neoplastic development. The rat tracheal explant system appears to be well suited for such studies. Carcinogen exposure of primary tracheal explants *in vitro* leads to the appearance of transformed cell lines (Steele *et al.*, 1977, 1979). Early-passage cells are often not tumorigenic, but late-passage cell lines are. In order to determine whether a change in antigen expression during neoplastic development occurs, it was necessary to show that tumorigenic tracheal epithelial cells transformed *in vitro* express antigens not present on untransformed cells, and to find the means to quantitate such antigens. While it has been established that tracheal tumours induced by carcinogenic polycyclic aromatic hydrocarbons *in vivo* express antigens capable of stimulating host responses (Jamasbi & Nettesheim, 1977*a*; Jamasbi *et al.*, 1978) there have been no reports on the immunogenicity of tracheal epithelial cell lines transformed *in vitro*. We report here that tumorigenic cell lines derived from rat tracheal explants exposed to N-methyl-N′-nitro-N-nitrosoguanidine (MNNG) are immunogenic in adult syngeneic rats. While both cellular and humoral responses can be detected, the antibody response will be most useful for studying the emergence of malignant cell populations. Such studies are reported below.

### MATERIALS AND METHODS

*Cell lines.*—The two tracheal cell lines used originated from tracheal explants which were exposed for 6 h on Days 3 and 6 of culture to 10 μg MNNG/ml in Waymouth's MB 752/1 medium (Marchok *et al.*, 1975, 1977). Explants were obtained from 10–12-week-old female Fischer-344 rats. After each carcinogen exposure, cells were incubated in complete medium (Waymouth's medium supplemented with 10% FCS, insulin, hydrocortisone, non-essential amino acids and fatty acids; see Marchok *et al.*, 1977). Primary cultures were established from explants and stable epithelial cell lines were obtained as described previously (Steele *et al.*, 1977). Cell lines were passaged weekly by trypsin dissociation as described by Steele *et al.* (1979).

Cell lines at various passage levels were tested for tumorigenicity in either immunosuppressed syngeneic rats or athymic BALB/c (*nu/nu*) mice. Rats were immunosuppressed (thymectomized and whole-body X-irradiated) as described by Jamasbi &Nettesheim (1977*b*), and were inoculated i.m. with $10^6$ viable cells in 0·2 ml Hanks's balanced salt solution (HBSS). Athymic BALB/c (*nu/nu*) mice were inoculated s.c. with $10^6$ cells in 0·1 ml HBSS. Animals were checked weekly for tumour development.

The two cell lines, 8-10-2 and 2-10-1, were used to immunize syngeneic rats and as cell targets to demonstrate host immune responses. For these purposes, cultures were propagated in complete Waymouth's media in which syngeneic F-344 rat serum (supplemented to 2%) was substituted for FCS for at least 2 passages before use.

*Induction of transplantation immunity.*—F-344 female rats (10–12 weeks old) were inoculated with tumorigenic passages of either 8-10-2 or 2-10-1 cells. Tumour-bearing legs were amputated when tumours were 3–5 cm in diameter (45–60 days for 2-10-1 and 30–50 days for 8-10-2). These animals were rechallenged (s.c.) 14–21 days later and subsequent tumour growth was surgically removed. This procedure continued until the animals rejected the challenge dose.

*Microcytotoxicity assay.*—The 20h microcytotoxicity assay (Takasugi & Klein, 1970) was used to measure spleen-cell cytotoxicity. Epithelial cell lines served as target cells and were propagated in complete Waymouth's media containing 2% isologous rat serum. Target cells were trypsinized and 100 viable cells in 0·1 ml were seeded in 96-well microtitre plates (Falcon No. 3034 Microtest plates). Only cultures with >90% viability were used. Nevertheless, plating efficiencies of tracheal cell lines in rat serum varied between experiments, though the number of cells attached after 24 h within a particular experiment was very consistent.

Spleen cells from rats which had rejected a tumorigenic cell challenge were used as the source of effector cells for microcytotoxicity assays, and as responder cells for *in vitro* sensitization experiments (Vose *et al.*, 1977). Rats which had resisted cell challenge were re-inoculated with $10^6$ cells, and their spleens were removed 7 days later. Spleens from non-immune F-344 rats served as controls. Spleen cells were separated from erythrocytes on 10ml Ficoll–Paque gradients (Pharmacia Fine Chemicals). Lymphoid cells were collected, washed ×3 and suspended in HBSS. Cell suspensions showed >98% viability by trypan-blue exclusion and contained 90% nucleated cells as determined by 0·1% crystal violet staining.

Effector spleen cells were added to target cells plated the previous day. Known numbers of effector cells were added to target cells in 0·1ml aliquots of RPMI 1640 medium supplemented with 10% FCS. Plates were incubated for 20 h at 37°C in 5% $CO_2$, washed ×3 with HBSS to remove non-adherent cells, and the remaining target cells fixed and stained with crystal violet. After air drying the target cells in each well were counted. Control cultures consisted of target cells incubated with either graded numbers of normal spleen cells or medium alone.

Spleen cells from immune rats were also co-cultivated *in vitro* with epithelial cell populations which had been lethally irradiated. Cells were X-irradiated as monolayer cultures in HBSS with 10 krad (250 rad/min). Irradiated cells were removed by trypsinization and showed viability of >90%. Normal lymphocytes were similarly irradiated but as a single-cell suspension.

Both irradiated ($2 \times 10^5$/ml) and responding ($2 \times 10^6$/ml) cell populations were suspended in RPMI 1640 medium containing 10% FCS, L-glutamine, antibiotics and $5 \times 10^{-5}$M 2-

mercaptoethanol (Cerottini *et al.*, 1974). One ml of each cell population was incubated in 12 × 75mm plastic tubes (Falcon No. 2054) for 6 days in a humidified 37°C incubator. To compensate for shifts in pH during incubation, the medium was buffered with 3·7 g/l sodium bicarbonate and incubated in 8% $CO_2$–air.

After co-cultivation, cells from each group were pooled, washed × 3 and the viability was determined. Responder spleen cells were used as effector cells in 20h microcytotoxicity assays as described above. Controls consisted of spleen cells incubated for 6 days with irradiated lymphocytes from normal F-344 rats.

*Antibody binding test* (*ABT*).—Target cells were grown as monolayer cultures on 10·5 × 22mm glass coverslips (Bellco Glass Co.). Directly before use, monolayer coverslips were chilled in cold HBSS, drained and floated cell-side down for 1 h at 4°C on 0·1 ml antiserum diluted in HBSS. Coverslips were washed in 2 changes of chilled HBSS to remove unbound antibody, and floated on 0·1 ml radiolabelled affinity-chromatography-purified, rabbit antibody to rat IgG (PARG) (Kennel & Feldman, 1976) for 1 h at 4°C (0·5 μg antibody protein per reaction). PARG was radiolabelled to specific activities of 5–10 × $10^5$ ct/min/μg with $Na^{125}I$ (New England Nuclear) using Chloramine T (McConahey & Dixon, 1966). Coverslips were washed in 3 changes of cold HBSS to remove unbound label, and cell-bound radioactivity was determined with a Searle Model 1185 γ-counter.

Controls consisted of monolayer cultures incubated with: (a) HBSS followed by labelled PARG and (b) cell monolayers incubated with diluted normal rat serum (NRS). In all cases, nonspecific binding (NRS control) was subtracted from experimental values before estimating the amount of labelled antibody protein specifically bound to target-cell monolayers.

*Fluorescent antibody tests.*—Coverslips of 8-10-2 or 2-10-1 cells or normal cells from primary tracheal outgrowths were prepared as described above. The cells were washed and incubated with normal or immune serum as described above for the ABT, except that fluorescein isothiocyanate-conjugated PARG was substituted for radioiodinated PARG. Cells were viewed on a Zeiss model microscope with epi-illumination.

### RESULTS

### *Development of tumorigenicity of tracheal cell lines*

As described previously (Steele *et al.*, 1979) exposure of primary tracheal explant cultures to MNNG *in vitro* produced stable epithelial cell lines. Tumorigenicity of the MNNG-induced tracheal cell lines was determined by inoculation at various passages of $10^6$ cells into immunosuppressed F-334 rats or BALB/c (*nu/nu*) mice (Table I). The time elapsing until the first subculture could be attempted was 256 days for the 8-10-2 cell line and 117 days for the 2-10-1 cell line. Cell Line 8-10-2 became tumorigenic between Passages 9 and 11, and Cell Line 2-10-1 between Passages 20 and 35. No differences in tumorigenic potential have been observed between the nude mouse xenografts and syngeneic rat transplantations.

I.m. inoculation of 2-10-1 cells (Passage 35) into immunosuppressed rats led to rapid growth of highly malignant squamous-cell carcinomas (Fig. 1). Inocu-

TABLE I.—*Development of tumorigenicity of MNNG-induced* tracheal epithelial cell lines with successive* in vitro *cell passages*

| Cell line | Days to first subculture† | Passage | Days† | Tumorigenicity‡ Iso-graft | Xeno-graft |
|---|---|---|---|---|---|
| 8-10-2 | 256 | 7 | 321 | 0/4 | — |
| | | 9 | 334 | 0/4 | 0/2 |
| | | 11 | 346 | 4/4 | — |
| | | 22 | 418 | — | 4/4 |
| | | 35 | 502 | 5/5 | — |
| 2-10-1 | 117 | 9 | 180 | — | 0/2 |
| | | 11 | 194 | — | 0/4 |
| | | 17 | 242 | 0/4 | — |
| | | 20 | 274 | 0/4 | — |
| | | 35 | 389 | 4/4 | 4/4 |
| | | 63 | 595 | 4/4 | 4/4 |

* Tracheal organ cultures were exposed twice for 6 h to 10 μg/ml MNNG.

† Number of days after last carcinogen exposure.

‡ Determined by inoculation of $10^6$ cells into immunosuppressed Fischer-344 rats (isograft) or athymic BALB/c (*nu/nu*) mice (xenograft).

FIG. 1.—Typical squamous carcinomas after inoculation of the 35th passage of the 2-10-1 cell line. H. & E. ×200.

FIG. 2.—Well-differentiated adenosquamous carcinoma with glandular structures filled with mucin-like material, and typical of the carcinomas after inoculation of the 11th passage of the 8-10-2 cell line. H. & E. ×200.

lation of Passage 11 8-10-2 led to growth of mixed adenosquamous carcinomas (Fig. 2).

*Induction of transplantation resistance to malignant tracheal cell lines*

To determine whether the malignant phases of the two epithelial cell lines were immunogenic, normal F-344 rats were repeatedly challenged with viable cells, as shown in Table II. Since cells grown in culture can adsorb antigenic determinants from the serum in culture medium (Embleton & Iype, 1978) all inoculations were made with cells grown in 2% isologous rat serum. Both 8-10-2 and 2-10-1 cell lines induced transplantation resistance (the ability of an animal to resist a tumorigenic inoculation) in some or all of the animals by the 3rd challenge. The number of resistant animals in each group increased progressively with repeated cell challenges.

TABLE II.—*Induction of transplantation resistance in syngeneic rats*

| Exp. No. | Cell line | No. of inocula | Tumour take* |
|---|---|---|---|
| 1 | 8-10-2 | 2 | 5/5 |
| | | 3 | 1/5 |
| | | 4 | 0/5 |
| 2 | 8-10-2 | 2 | 5/5 |
| | | 3 | 4/5 |
| | | 4 | 2/5 |
| | | 5 | 1/5 |
| 3 | 2-10-1 | 2 | 5/5 |
| | | 3 | 0/5 |
| 4 | 2-10-1 | 2 | 5/5 |
| | | 3 | 3/5 |
| | | 4 | 0/5 |

* Number of tumour-bearing rats/rats tested.

*Induction of cell-mediated immunity*

Spleen cells from rats showing transplantation resistance to either 8-10-2 or 2-10-1 lines, or from unimmunized controls, were tested for cytotoxic reactivity against neoplastic cell lines or primary tracheal outgrowths. As is shown in Table III, 8-10-2-immune spleen cells caused a significantly greater reduction in neoplastic target cells than non-immune spleen cells. 2-10-1-immune spleen cells showed moderate to strong cytotoxicity for both target cell lines. While a linear spleen-cell dose–response relationship could not be demonstrated, in general, lower numbers of immune cells produced less cytotoxicity.

Primary tracheal outgrowths which cannot be propagated *in vitro* have very low plating efficiencies when trypsinized and used as cell targets (Table III, Exp. 4). However, there was negligible cytotoxicity of 8-10-2-immune spleen cells towards these untransformed cell targets, even at higher effector:target cell ratios (30,000 effector spleen cells per well).

In order to test more fully for the presence of antigen-bearing cells among normal or transformed epithelial cells (Engers & MacDonald, 1976) immune spleen cells were co-cultivated for 6 days *in vitro* with primary tracheal-outgrowth cultures or neoplastic epithelial cell lines before 20h microcytotoxicity testing. As shown in Table IV, significant reactivity for the immunizing target cell was obtained when immune spleen cells were co-cultivated with either of the neoplastic cell lines. No cytotoxicity of immune spleen cells for neoplastic target cells was detected after co-cultivation with normal tracheal outgrowths. Residual cytotoxicity of immune spleen cells was also lost during 6-day co-cultivation with irradiated lymphocytes, indicating that antigen-bearing cells must be present to maintain the cytotoxic lymphocytes. No significant differences were detected between target-cell numbers in buffer control and normal-lymphocyte control, with one exception, where an increase in number of target cells (expressed as a negative response) was observed (Table IV).

*Detection of circulating antibody*

Sera obtained from immune rats were first tested for antibody activity against 8-10-2 and 2-10-1 cell lines by the ABT. Fig. 3 compares binding of sera from 2-10-1- or 8-10-2-immune rats to 2-10-1

TABLE III.—*Microcytotoxicity assay demonstrating reactivity of spleen cells from immune rats to epithelial cell lines* 2-10-1 *and* 8-10-2

| | | | Spleen cells/well | | | | | | | |
|---|---|---|---|---|---|---|---|---|---|---|
| | | | 30,000 | | 15,000 | | 7500 | | 3700 | |
| Expt | Spleen-cell source† | Target cells | Cells/ well‡ | % Red.§ | Cells/ well | % Red. | Cells/ well | % Red. | Cells/ well | % Red. |
| 1 | 8-10-2-immune | 8-10-2 | 76 ± 4 | 39** | 89 ± 6 | 20** | 95 ± 7 | 25** | 99 ± 9 | 15* |
| | | 2-10-1 | 67 ± 7 | 26* | 59 ± 13 | 30** | 77 ± 6 | 32** | 109 ± 5 | −3 |
| | Normal | 8-10-2 | 124 ± 3 | | 111 ± 11 | | 124 ± 12 | | 117 ± 5 | |
| | | 2-10-1 | 91 ± 14 | | 85 ± 3 | | 114 ± 5 | | 106 ± 11 | |
| 2 | 8-10-2-immune | 8-10-2 | 23 ± 2 | 38** | 27 ± 2 | 18* | 29 ± 2 | 22* | 30 ± 2 | 15 |
| | | 2-10-1 | 20 ± 2 | 26* | 18 ± 4 | 28* | 23 ± 2 | 32** | 33 ± 2 | −3 |
| | | Normal outgrowth†† | 16 ± 4 | 11 | 18 ± 3 | −6 | 22 ± 3 | −5 | ND | |
| | Normal | 8-10-2 | 37 ± 2 | | 33 ± 5 | | 37 ± 3 | | 35 ± 2 | |
| | | 2-10-1 | 27 ± 5 | | 25 ± 4 | | 34 ± 2 | | 32 ± 3 | |
| | | Normal outgrowth | 18 ± 2 | | 17 ± 2 | | 21 ± 2 | | ND | |
| 3 | 2-10-1-immune | 2-10-1 | 68 ± 7 | 36** | 59 ± 13 | 48** | 77 ± 6 | 8 | 109 ± 5 | −36 |
| | Normal | 2-10-1 | 106 ± 11 | | 114 ± 5 | | 84 ± 3 | | 80 ± 6 | |
| 4 | 2-10-1-immune | 2-10-1 | 119 ± 8 | 50** | 138 ± 12 | 39** | 145 ± 13 | 37** | 180 ± 9 | 21** |
| | | 8-10-2 | 42 ± 2 | 64** | 45 ± 2 | 55** | 47 ± 5 | 57** | 74 ± 5 | 33** |
| | Normal | 2-10-1 | 236 ± 9 | | 227 ± 10 | | 230 ± 10 | | 228 ± 12 | |
| | | 8-10-2 | 117 ± 6 | | 100 ± 9 | | 114 ± 4 | | 111 ± 7 | |

† Spleen cells originated from syngeneic rats resistant to Cell Lines 8-10-2 or 2-10-1 or from normal rats. There was no significant difference in number of target cells surviving, between wells without spleen cells and those incubated with normal spleen cells.

‡ Average number of cells surviving per well ± s.e.

§ Decrease in target-cell number after exposure to immune spleen cells compared to those exposed to normal spleen cells. **P* < 0·05 or ***P* < 0·01 by *t* test.

†† From untreated F-344 tracheal explants.

ND = Not done.

or 8-10-2 cell monolayers, respectively. Normal F-344 rat serum was used at appropriate dilutions to determine non-specific binding, and these values (never > 15 ng) were subtracted from experimental results. Antibody binding is easily detected even at 1/1000 dilutions in sera from these immune rats.

The specificity of antibody binding was determined by absorption of immune sera. Antibody binding of anti-2-10-1 serum (diluted 1:80) was compared to binding after absorption with either normal lung homogenates or tumour homogenates from 2-10-1 cells propagated *in vivo* (Fig. 4A). Absorption with normal-lung homogenates did not significantly reduce anti-2-10-1 activity for 2-10-1 monolayers. In contrast, absorption of 2-10-1-immune serum with 2-10-1 cell homogenates removed 94% of the binding activity. Analogous results were obtained with 8-10-2-immune serum (Fig. 4B). The ability of homologous tumour-cell absorptions to remove antibody activity of immune sera provided strong evidence that binding activity of such antisera were not directed against foetal calf serum or other *in vitro* components.

Cross-reactivity between the two malignant cell lines was demonstrated by serial absorptions of serum obtained from transplantation-resistant rats. Fig. 5A shows the residual binding activity of anti-2-10-1 serum on 2-10-1 cell monolayers after absorption with normal tissue or tumour-cell homogenates. Absorption with normal-tissue homogenates removed less than 15% of the original antibody activity, whereas 82% of the original antibody activity was removed by 3 absorptions with 8-10-2 tumour homogenates. Two absorptions using homologous 2-10-1 tumour homogenates removed > 98% of

TABLE IV.—*Microcytotoxicity assay of spleen cells from immune rats, after 6-day co-cultivation* in vitro *with lethally irradiated stimulating cells, to epithelial Cell Lines* 2-10-1 *and* 8-10-2

| | | | Spleen cells/well | | | | | |
|---|---|---|---|---|---|---|---|---|
| | | | 30,000 | | 15,000 | | 7500 | |
| Target cell | Responder spleen cell | X-irradiated stimulating cell | Cells/ well‡ | % Red.§ | Cells well | % Red. | Cells/ well | % Red. |
| 8-10-2 | 8-10-2-immune | 8-10-2 | 31±5 | 41** | 38±5 | 37** | 54±6 | 18* |
| | | 2-10-1 | 40±2 | 24* | 50±8 | 18* | 46±3 | 30* |
| | | Normal outgrowths | 55±9 | −4 | 66±9 | 8 | 66±6 | 0 |
| | | Lymphocytes† | 49±9 | 8 | 54±10 | 11 | 45±2 | 32* |
| | Buffer†† | | 53±5 | | 61±5 | | 66±9 | |
| 8-10-2 | 8-10-2-immune | 8-10-2 | 43±3 | 49** | 50±6 | 44** | ND | |
| | | 2-10-1 | 55±3 | 34** | 67±8 | 25** | ND | |
| | | Normal outgrowths | 76±7 | 9 | 86±5 | 3 | ND | |
| | | Lymphocytes | ND | | 85±8 | 4 | ND | |
| | Buffer | | 84±5 | | 89±7 | | ND | |
| 2-10-1 | 2-10-1-immune | 2-10-1 | ND | | 39±7 | 42** | 31±1 | 54** |
| | | 8-10-2 | ND | | 43±10 | 36** | 25±5 | 61** |
| | | Normal outgrowths | ND | | 63±10 | 6 | ND | |
| | | Lymphocytes | ND | | 90±5 | −34 | 85±6 | −24 |
| | Buffer | | ND | | 67±7 | | 67±11 | |

† Normal F-344 rat lymphocytes.
‡ Average number of target cells per well ± s.e.
§ Percentage cell reduction of target cells exposed to spleen cells co-cultivated with irradiated cell populations as compared to target cells incubated for 6 days in buffer. $*P < 0{\cdot}05$ or $**P < 0{\cdot}01$ ($t$ test).
†† Target cells incubated without spleen cells or with spleen cells co-cultivated with normal rat lymphocytes. ND = Not done.

FIG. 3.—Antibody binding test (ABT) of serum from F-344 rats resistant to tumorigenic 8-10-2 or 2-10-1 cell challenge. ○—○, reactivity of 2-10-1 immune serum for cultured 2-10-1 cells. ●—●, reactivity of 8-10-2 immune serum for cultured 8-10-2 cells. Nonspecific binding was determined for each serum dilution by substituting normal rat serum for immune serum, and subtracted from experimental values.

FIG. 4.—Antibody binding of serum from immune rats after absorption with normal F-344 lung or tumour homogenates. A. Serum from 2-10-1 immune rats was diluted 1:80 and absorbed overnight with either normal F-344 lung or with 2-10-1 tumour homogenates (+) and residual antibody binding determined on 2-10-1 cell monolayers by ABT. Binding results are compared to an aliquot of antiserum similarly treated but not absorbed (−). B. Comparable tests with 8-10-2 cells. $t$ test for significance. NS = not significant.

the antibody activity. Analogous results were obtained in the reciprocal experiment on anti-8-10-2 serum (Fig. 5B).

The ability of 8-10-2- or 2-10-1-immune rat antibody to bind specifically to neoplastic epithelial cell lines was also demonstrated by indirect immunofluorescence.

FIG. 5.—Antibody binding of serum from immune rats after serial absorptions with normal F-344 rat tissue or tumour homogenates. Antisera were absorbed as described in Fig. 4, separated from absorbing homogenates by centrifugation (800 *g* for 10 min), and absorption repeated. A. Residual antibody activity on 2-10-1 cell monolayers after serial absorptions of 2-10-1-immune serum. B. Residual antibody activity of absorbed anti-8-10-2 serum. Results (% of control binding) represent binding (ct/min) of the absorbed sample divided by binding of a non-absorbed portion of the same antiserum (×100). Antisera were absorbed with 2-10-1 (●), 8-10-2 (○), normal rat brain (△), or liver (▲) homogenates.

TABLE V.—*Indirect immunofluorescence assay on antibody binding in immune-rat sera for normal tracheal outgrowths or neoplastic cell lines*

| Serum source | Serum dilution | Fluorescing cells* 2-10-1 | 8-10-2 | Primary outgrowths |
|---|---|---|---|---|
| Immune to 2-10-1 | 1/25 | + | ND | – |
| | 1/50 | + | ND | – |
| Normal rat | 1/25 | – | ND | – |
| | 1/50 | – | ND | – |
| Immune to 8-10-2 | 1/25 | + | + | – |
| | 1/50 | + | + | – |
| Normal rat | 1/25 | – | – | – |
| | 1/50 | – | – | – |

* Cells were stained in the cold with FITC-labelled anti-rat IgG diluted 1:100 in HBSS with 1% BSA. Staining of cells was assessed subjectively as: – (<5% of the cells fluorescing) or + (>5% of the cells fluorescing).

Both anti-8-10-2 and anti-2-10-1 sera were positive on homologous target-cell monolayers. In addition, 8-10-2-immune antiserum stained the surface membranes of both neoplastic cell lines (Table V) indicating cross-reactive determinants. About 50% of the neoplastic cells were stained, whereas nonspecific fluorescence, as determined using NRS as controls, never exceeded 5% of the cells. Under these conditions, neither 8-10-2- nor 2-10-1-immune rat sera demonstrated staining on primary tracheal outgrowths.

*Endogenous C-type virus activity*

Cell lines used in these studies were analysed for C-type viral gene products. First, lysates of cells grown to confluency were analysed for the major core protein p30 using an interspecific radioimmunoassay (Strand & August, 1974; Kennel & Tennant, 1979). This type of assay has been shown to detect p30s from rat, mouse, and cat viruses (Strand & August, 1974; Rasheed *et al.*, 1976). Cell lysates from confluent 100mm plates of 8-10-2 or 2-10-1 cells ($\sim 5 \times 10^6$ cells) contained less than 5 ng of p30, which was 50–100 times less than in virus-infected fibroblast cultures. Secondly, putative virus pellets, concentrated 100-fold from culture fluids, were analysed for reverse-transcriptase activity by the method of Ross *et al.* (1971). Neither 8-10-2 nor 2-10-1 cells demonstrated enzyme levels above control (normal fibroblast) values, whereas mouse virus isolated from mouse fibroblasts and baboon endogenous virus isolated from canine thymus cells had enzyme activities at least 20 times background.

Finally, syngeneic antisera to 2-10-1 and 8-10-2 cells were analysed for antibody to gp70 by a sensitive antibody-binding-capacity assay (Kennel, 1976). Of 8 sera tested, none demonstrated significant binding of 1 ng of $^{125}I$ gp70 (<2%), while control antisera diluted 1000-fold bound 82% of the labelled protein. Thus there was no detectable expression of C-type viral genes in the rat tracheal-cell lines, which are unlikely to be factors in the immune response of syngeneic animals.

### DISCUSSION

These studies have demonstrated that tumorigenic passages of epithelial cell lines derived from rat tracheal explants transformed *in vitro* by MNNG acquire antigens capable of stimulating syngeneic immunological responses. The two transformed epithelial cell lines used in this report were shown to be immunogenic by induction of transplantation resistance against tumorigenic inocula. Once transplantation immunity was established, both humoral and cell-mediated immune responses were evident. Previous studies using *in vivo* carcinogen-induced pulmonary adenomas of mice (Prehn, 1962; Pasternak *et al.*, 1966) and tracheal and pulmonary squamous carcinomas of rats (Jamasbi *et al.*, 1978) have suggested that respiratory-tract tumours are either poorly immunogenic or incapable of eliciting any type of syngeneic immune response. Immunogenicity of both *in vitro* carcinogen-induced cell lines was weak, but demonstrable, and is consistent with findings from *in vivo* chemical induction.

Cell-mediated immune responses were demonstrated with both primary effector spleen cells (freshly harvested) or after *in vitro* co-cultivation with either neoplastic cell line before microcytotoxicity assay (secondary effector cells). Reactivity of primary spleen effector cells was limited to the neoplastic cell lines, and spleen-cell reactivity against untransformed tracheal outgrowths was not detected. Furthermore, reactivity of secondary effector spleen cells for neoplastic cell targets was maintained only by co-cultivation *in vitro* with the neoplastic cell lines. Normal tracheal outgrowths were incapable of generating secondary effector cells *in vitro*. These results support the conclusion that immune spleen cells were reacting with tumour-cell antigens not present in the normal cell population

We have also demonstrated that sera from immune rats contained antibody directed at cell-surface antigens on neoplastic epithelial cell lines. Anti-tumour antibody to rat respiratory-tract carcinomas induced *in vivo* (Jamasbi & Nettesheim, 1977*a*; Jamasbi *et al.*, 1978) and to human respiratory-tract carcinomas (Gorny *et al.*, 1979; Sofen & O'Toole, 1978) has been reported. In all cases, some activity was also detected against normal respiratory tissue. We were not able to detect significant antibody activity against normal respiratory-tract tissues by antiserum absorptions with lung homogenates. Indirect immunofluorescence, which supported the findings of the ABT, also failed to reveal any evidence of antibody activity for normal primary outgrowths.

The cross-reactivities of 8-10-2 and 2-10-1 cell lines have not been evaluated *in vivo*. However, common tumour-rejection antigens were found among several benzo(a)pyrene or dimethylbenz(a)-anthracene respiratory-tract carcinomas induced *in vivo* in F-344 rats (Jamasbi & Nettesheim, 1977*b*). The cross-reactivity obtained *in vitro* using spleen cells from tumour-immune animals appears to be specific for the transformed cell lines, since no reactivity was found for untransformed tracheal epithelial cells. Furthermore, it is unlikely that cross-reactivity between the two epithelial cell lines was due to an endogenous viral genome activated during *in vitro* carcinogenesis (Rasheed, 1979). No evidence of reverse-transcriptase activity was found in these cell lines, and binding of tumour-immune or tumour-bearing sera to the gp70 macromolecule of murine leukaemia virus was not demonstrable.

The presence of common antigens on the surface of 8-10-2 and 2-10-1 cell lines was also demonstrated by absorption experiments. Absorption of 2-10-1-immune serum with 8-10-2 homogenates decreased antibody binding to plateau levels of about 80% of the original activity. Quantitative absorption tests with enhanced sensitivity will have to be used to determine whether specificities for a single tumour type are also present in tumour-immune serum.

Rat tracheal-cell explants can be transformed *in vitro* by carcinogens to generate epithelial cell lines (Steele *et al.*, 1977, 1979) which upon continued *in vitro* propagation become tumorigenic. In this report, we have used the syngeneic immune response to identify cell-surface antigens on the tumorigenic cell populations. Such antigens detected on transformed cell lines must have been acquired as a result of neoplastic transformation, since it was shown that untransformed cell outgrowths lack detectable amounts of these neo-antigens. It is at present not known whether the appearance of neo-antigens on chemically altered cells is an essential feature of oncogenesis, or whether it is merely a byproduct of the interaction between the carcinogen and the cells.

This research was sponsored jointly by the National Institute of Environmental Health Sciences under Interagency Agreement 222-YO1-ES-20041 and the Office of Health and Environmental Research, U.S. Department of Energy, under Contract W-7405-eng-26 with the Union Carbide Corporation.

### REFERENCES

BORENFREUND, E., HIGGINS, P. J., STEINGLASS, M. & BENDICH, A. (1975) Properties and malignant transformation of established rat liver parenchymal cells in culture. *J. Natl Cancer Inst.*, **55**, 375.

COLBURN, N. H., VORDER BRUEGGE, W. F., BATES, J. R. & 4 others (1978) Correlation of anchorage-independent growth with tumorigenicity of chemically transformed mouse epidermal cells. *Cancer Res.*, **38**, 624.

CEROTTINI, J.-C., ENGERS, H. D., MACDONALD, H. R. & BRUNNER, K. T. (1974) Generation of cytotoxic T lymphocytes *in vitro*. I. Response of normal and immune mouse spleen cells in mixed leukocyte cultures. *J. Exp. Med.*, **140**, 703.

EMBLETON, M. J. & IYPE, P. T. (1978) Surface antigens of rat liver epithelial cells grown in medium containing foetal bovine serum. *Br. J. Cancer*, **38**, 456.

ENGERS, H. D. & MACDONALD, R. H. (1976) The generation, detection and characterization of cytotoxic T lymphocyte activity *in vitro*. *Scand. J. Immunol.*, **5**, 135.

GÓRNY, M. K., JEŻEWSKA, E., KRZYŚKO, R., STARWARZ, M. & ŻEROMSKI, J. (1979) Anti-tumor antibodies in lung cancer patients: An immunofluorescence study using various indicator cells. *Neoplasma*, **26**, 6.

HASHIMOTO, Y. & KITAGAWA, H. S. (1975) *in vitro* neoplastic transformation of epithelial cells of rat urinary bladder by nitrosamine. *Nature*, **252**, 497.

JAMASBI, R. J. & NETTESHEIM, P. (1977*a*) Demonstration of cross-reacting tumor rejection antigens in chemically induced respiratory tract carcinomas in rats. *Cancer Res.*, **37**, 4059.

JAMASBI, R. J. & NETTESHEIM, P. (1977*b*) Non-immunological enhancement of tumour transplantability in X-irradiated host animals. *Br. J. Cancer*, **36**, 723.

JAMASBI, R. J., NETTESHEIM, P. & KENNEL, S. J. (1978) Demonstration of cellular and humoral immunity to transplantable carcinoma derived from the respiratory tract of rats. *Cancer Res.*, **38**, 261.

KENNEL, S. J. (1976) Purification of glycoprotein from mouse ascites fluid by immunoaffinity chromatography which is related to the major glycoprotein of murine leukemia viruses: Immunologic and structural comparison with purified viral glycoproteins. *J. Biol. Chem.*, **251**, 6197.

KENNEL, S. J. & FELDMAN, J. D. (1976) Distribution of viral glycoproteins gp69/71 on cell surfaces of producer and non-producer cells. *Cancer Res.*, **36**, 200.

KENNEL, S. J. & TENNANT, R. W. (1979) Analysis of proteins of mouse sarcoma pseudotype viruses: Type-specific radioimmunoassays for ecotropic virus p30's. *J. Virol.*, **30**, 729.

KNOWLES, M. A. & FRANKS, L. M. (1977) Stages in neoplastic transformation of adult epithelial cells by 7,12-dimethylbenz(a)anthracene *in vitro*. *Cancer Res.*, **38**, 3917.

MCCONAHEY, P. J. & DIXON, F. J. (1966) A method to trace iodination of proteins for immunological studies. *Int. Arch. Allergy Appl. Immunol.*, **29**, 185.

MARCHOK, A. C., CONE, V. & NETTESHEIM, P. (1975) Induction of squamous metaplasia (vitamin A deficiency) and hypersecretory activity in tracheal organ cultures. *Lab. Invest.*, **33**, 451.

MARCHOK, A. C., RHOTON, J., GRIESEMER, R. & NETTESHEIM, P. (1977) Increased *in vitro* growth capacity of tracheal epithelium exposed *in vivo* to 7,12-dimethylbenz(a)anthracene. *Cancer Res.*, **37**, 1811.

PASTERNAK, G., HOFFMAN, F. & GRAFFI, A. (1966) Growth of diethylnitrosamine-induced lung tumors in syngeneic mice specifically pretreated with X-ray killed tumor tissue. *Folia Biol. (Praha)*, **12**, 299.

PREHN, R. T. (1962) Specific isoantigenicities among chemically induced tumors. *Ann. N.Y. Acad. Sci.*, **101**, 107.

RASHEED, S., BRUSZWESKI, J., RONGEY, R. W., ROY-BURMAN, P., CHARMAN, A. P. & GARDNER, M. B. (1976) Spontaneous release of endogenous ecotropic type C virus from rat embryo cultures. *J. Virol.*, **18**, 799.

RASHEED, S. (1979) Endogenous virogenes and oncogenes in rat-cell transformation: A new model system. Cold Spring Harbor Symp. *Quant. Biol.*, **44**, 793.

ROSS, J., SCOLNICK, E. M., TODARO, G. J. & AARONSON, S. A. (1971) Separation of murine cellular and murine leukemia virus DNA polymerases. *Nature*, **231**, 163.

SOFEN, H. & O'TOOLE, C. (1978) Anti-squamous tumor antibodies in patients with squamous cell carcinoma. *Cancer Res.*, **38**, 199.

STEELE, V. E., MARCHOK, A. C. & NETTESHEIM, P. (1977) Transformation of tracheal epithelium exposed *in vitro* to N-methyl-N′-nitro-N-nitrosoguanidine (MNNG). *Int. J. Cancer*, **20**, 234.

STEELE, V. E., MARCHOK, A. C. & NETTESHEIM, P. (1979) Oncogenic transformation in epithelial cell lines derived from tracheal explants exposed *in vitro* to N-methyl-N′-nitro-N-nitrosoguanidine. *Cancer Res.*, **39**, 3805.

STRAND, M. & AUGUST, J. T. (1974) Structural proteins of mammalian oncogenic RNA viruses: Multiple antigenic determinants of the major internal protein and envelope glycoprotein. *J. Virol.*, **13**, 171.

TAKASUGI, M. & KLEIN, E. (1970) A microassay for cell-mediated immunity. *Transplantation*, **9**, 219.

VOSE, B. M., VANKY, F. & KLEIN, E. (1977) Lymphocyte cytotoxicity against autologous tumor biopsy cells in humans. *Int. J. Cancer*, **20**, 512.